# Development of an Offline, Open-Source, Electronic Health Record System for Refugee Care

**DOI:** 10.3389/fdgth.2022.847002

**Published:** 2022-03-14

**Authors:** Tobias Brotherton, Samuel Brotherton, Henry Ashworth, Adesh Kadambi, Hassaan Ebrahim, Senan Ebrahim

**Affiliations:** ^1^Hikma Health, San Jose, CA, United States; ^2^Harvard Medical School, Boston, MA, United States; ^3^University of Toronto, Toronto, ON, Canada; ^4^Harvard University John F. Kennedy School of Government, Cambridge, MA, United States

**Keywords:** electronic health record, mHealth, refugee, displaced population, low resource

## Abstract

While electronic health records (EHRs) have been shown to be effective in improving patient care in low-resource settings, there are still barriers to implementing them, including adaptability, usability, and sustainability. Taking a user-centered design process we developed the Hikma Health EHR for low resourced clinics caring for displaced populations. This EHR was built using React Native and Typescript that sync to a Python backend repository which is deployed on Google Cloud SQL. To date the Hikma Health EHR has been deployed for 26,000 patients. The positive impacts of the system reported by clinician users are 3-fold: (1) improved continuity of care; (2) improved visualization of clinical data; and (3) improved efficiency, resulting in a higher volume of patients being treated. While further development is needed, our open-source model will allow any organization to modify this system to meet their clinical and administrative needs.

## Introduction

The estimated displaced population worldwide by United Nations High Commissioner for Refugees (UNHCR) in 2019 was overs 80 million, a number that has doubled in the last decade (1). The number of displaced people is expected to continue to rise due to extreme weather events and conflict (1, 2). While the underlying factors that are causing the displacement of people need to be addressed, systems that provide healthcare to these populations also need to be improved.

Beyond the initial trauma from displacement itself, displaced populations are uniquely vulnerable to continual health threats from perpetual violence, exacerbation of chronic health conditions, food insecurity, and infectious diseases (2–4). Delivering healthcare to displaced populations that meets these challenges requires a high level of resources and system integration, however resources for such populations are often limited. In the past healthcare for refugees has relied on refugee camp-based care and limited services sponsored by host country governments (5, 6). Due to limited resources, refugee clinics often operate without formal systems for recording patient information, further amplifying the challenges of irregular care that can impact the quality of care, such as the management of chronic diseases and medications (7). The overall outcome from the current healthcare systems in place often increase patient burden and perpetuate poor health outcomes (8). Therefore, there is an urgent need to develop specific systems solutions designed for this specific population which meet the constraints of the settings in which they live.

Electronic health records (EHR) have shown to be a promising solution in this setting (4, 9–11). While traditional EHRs have been built in and for high-resource settings and mainly designed to be optimized for billing, there are a number of solutions being developed for low-resource settings and displaced populations, though to date these are few in number (9, 11, 12). Two EHRs that were built from the ground up within specific clinics were shown to improve chronic disease care for Syrian and Palestinian refugees (8, 9, 11, 12). However, these EHRs are single site solutions and cannot easily be implemented across multiple organizations. Another EHR solution, Sijilli, is a cloud-based mobile EHR developed in partnership between Epic Systems Corporation and the American University of Beirut (13). Sijilli offers a number of creative solutions for displaced people, including a personal flash drive with their medical record which is also backed up to a cloud data center, that is password protected. While there are multiple data security elements and it is designed to be scalable, the closed source nature of Sijilli EHR means it cannot be simply modified to meet an organizations' needs. Therefore, there is a need to create a more adaptable, equitable, and cost-effective EHR solution, specifically designed for displaced populations, that can be utilized by any organization worldwide.

Hikma Health is a 501(c)3 non-profit specifically founded to meet this need. By utilizing a human-centered design process we have designed a free and open-source EHR specifically for displaced populations (14). In the following paper we discuss the development and innovations of the Hikma Health system followed by a description of the successes in challenges so far in implementing it in the care of displaced populations.

## Methods

### User Experience Design

The design process began with preliminary research interviews with patients and providers in various displaced population healthcare settings, including mobile clinics, multi-specialty clinics, and hospitals in developing regions including areas of the Middle East. We interviewed a total of 12 providers for an average of 60 min per provider in an in-person interview. Providers were clinical staff members at non-profit clinical organizations delivering care for Syrian refugee patients in Jordan, Lebanon, Turkey, and Greece. Providers were contacted for brief 15–30 min follow-up interviews by phone. Interviews were conducted by authors SE and HE in English and Arabic. Responses to interview questions were audio recorded by the interviewers with consent of the providers with notes recorded simultaneously by the interviewers. The design process involved a framework analysis to synthesize provider input as previously described by the authors (15).

Research questions we explored including:

- How are physicians on the ground currently managing refugee care?
- How are they recording, sharing, and reviewing health data about their patients?
- Where are the current resources falling short of their expectations?
- How would a new software system be able to empower them as users to deliver the highest quality of care possible?

We first identified gaps in documentation, including specific needs to enhance workflows and resource constraints across the settings we interviewed. From our findings, we built prototypes with design tools, like InVision and Figma, to bring concepts to a fidelity where they were first tested by volunteer physicians internally and then to a group of users from partner organizations to gather feedback. Through this prototype testing process, we synthesized our learnings and identified three essential features for an effective digital health system in low-resource settings: modular workflows, multilingual interfaces, and offline-first capabilities. Although first generation EHRs were limited by technical constraints (16), modern EHRs like the Hikma Health system incorporate modular workflows. Modular workflows are defined as custom documentation workflows based on specific clinical protocols that can be easily added by the user based on the needs of a particular clinical visit. This modularity is essential to appropriately provisioning the application based on local clinical practice, such as consulting specialty care services or tracking prescriptions over time (17). After building a product to meet these core needs, the Hikma Health system was launched with clinical organizations providing primary care services in low-resource settings. We for continued product design iteration, based on feedback shared in both verbal and written form by users based on from daily clinical use at our partner clinics. Features requested by users were prioritized based on anticipated impact on clinical workflow and ease of implementation. We regularly updated the functionality of the software based on this user feedback process.

### Mobile Application Front End

**Figure 1:**
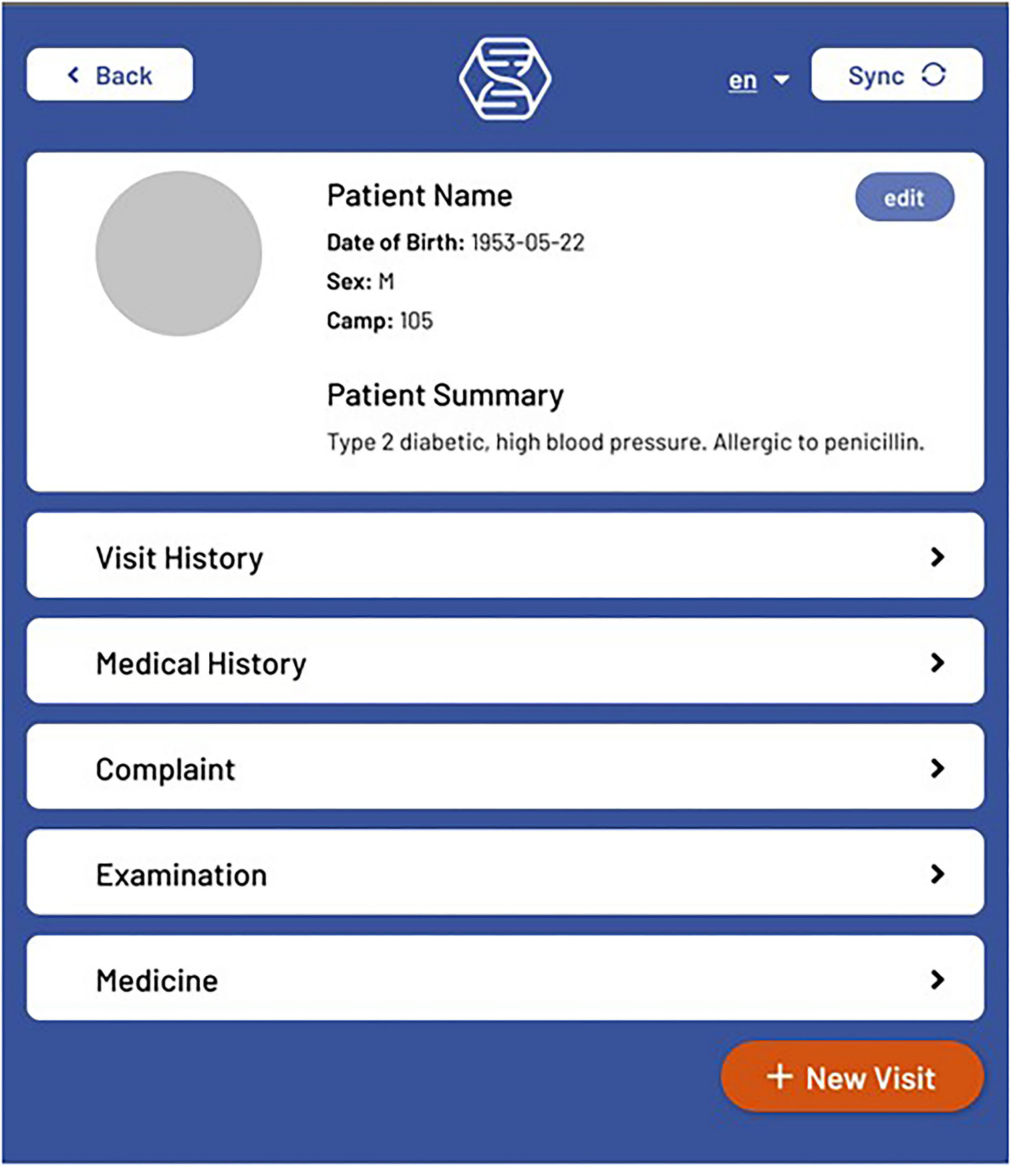
The Hikma Health Patient View screen where a summary of all patient information can be viewed.

**Figure 2:**
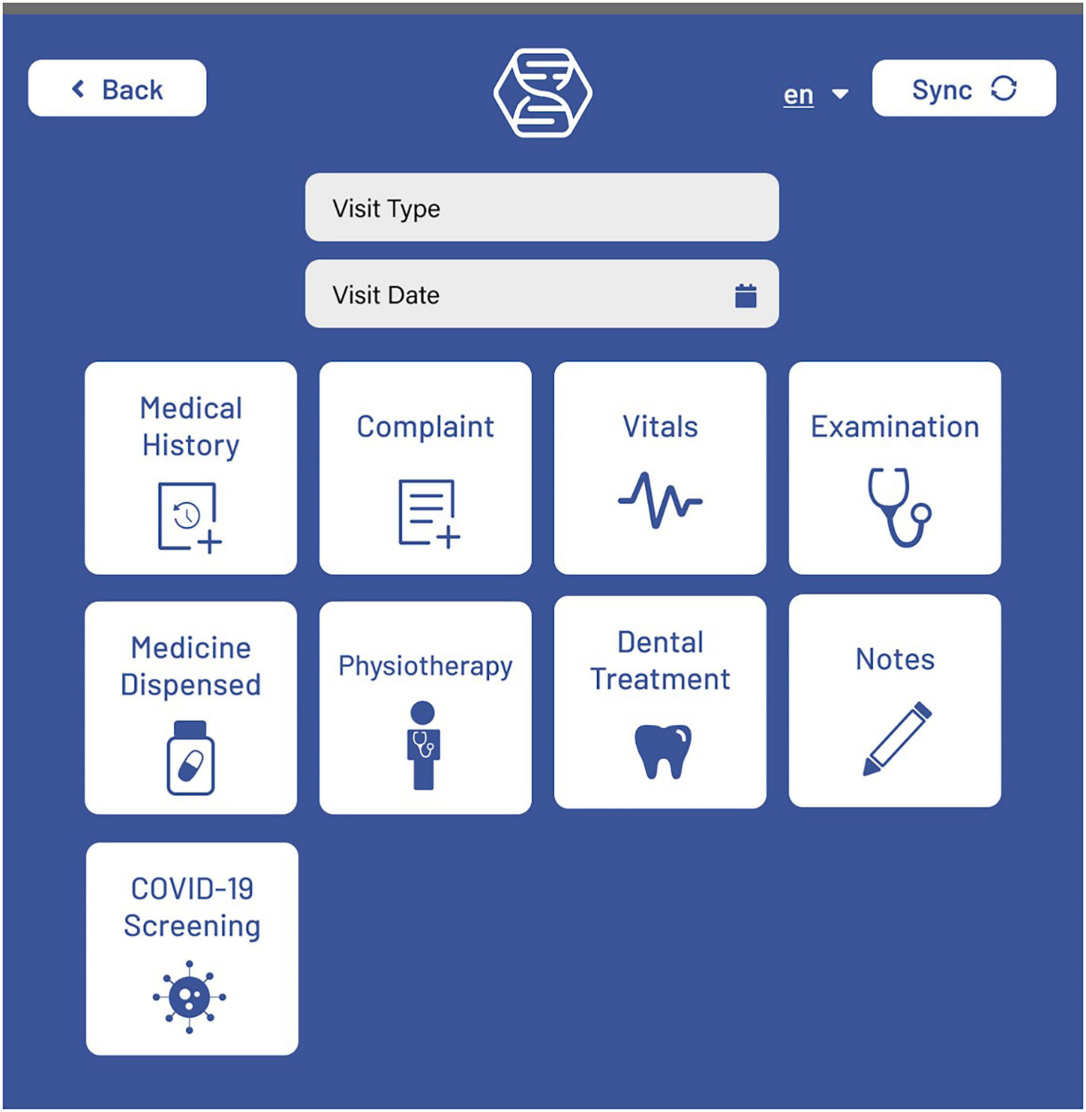
The Hikma Health New Patient screen where a number of modular work flows can be selected.

*The front end* for the mobile application is built using React Native and Typescript. Screen navigation is managed with React Stack Navigation. Display text for all screens is saved in English, Spanish, and Arabic. Most screens have a language toggle where the preferred language can be selected. After logging in, the user is directed to a Patient List screen, where new patients can be added, patient data can be synced, and patients can be searched for using a variety of search filters. Selecting a patient takes the user to the Patient View (Figure 1) screen, where some basic user information is displayed. Patient visits can be added, which opens a New Visit (Figure 2) screen that shows the different options for visit events. Visit event types differ based on the clinic's needs, but some common events are vital signs, medical evaluation, dispensed medications, notes, lab tests, etc.

From the Patient View screen, patient details and patient summary events can also be created to add further pertinent patient information. The basic patient information fields and patient photos can also be edited from this screen. Also, the Patient Snapshot on this screen provides a customizable subset of event information for all previous patient visits that is displayed by event type. The Visit History screen can be opened for a given patient, where previous visits can be viewed, deleted or edited by adding new visit events. From the Visit History screen, all events are displayed that occurred for the selected visit. During the onboarding process, clinics are provided with a manuscript for the full stack implementation of creating and editing visit events, and the optional addition of these events to the Patient Snapshot.

### Admin Application Front End

The mobile application is accompanied by a web based *admin application* used for user management and exporting patient data. This is a simple application built with React and Material Design. The Home page of the admin app features a list of users where passwords can be updated and users can be added/deleted. There is a link to a page that displays a list of patients, where patient information can be exported as a spreadsheet for each individual patient, or for all patients of the clinic. Rows in the exported spreadsheet correspond to visits, with the visit event fields being columns. The generation of the spreadsheet for all patients takes some time, so this is executed by a Kubernetes cronjob, and stored in a GCS bucket. The frequency of this report generation is fully customizable by the clinic. When the spreadsheet for all patients is requested in the admin app, the latest generated spreadsheet is retrieved from the bucket and automatically downloads on the user's computer.

### Backend Architecture

**Figure 3:**
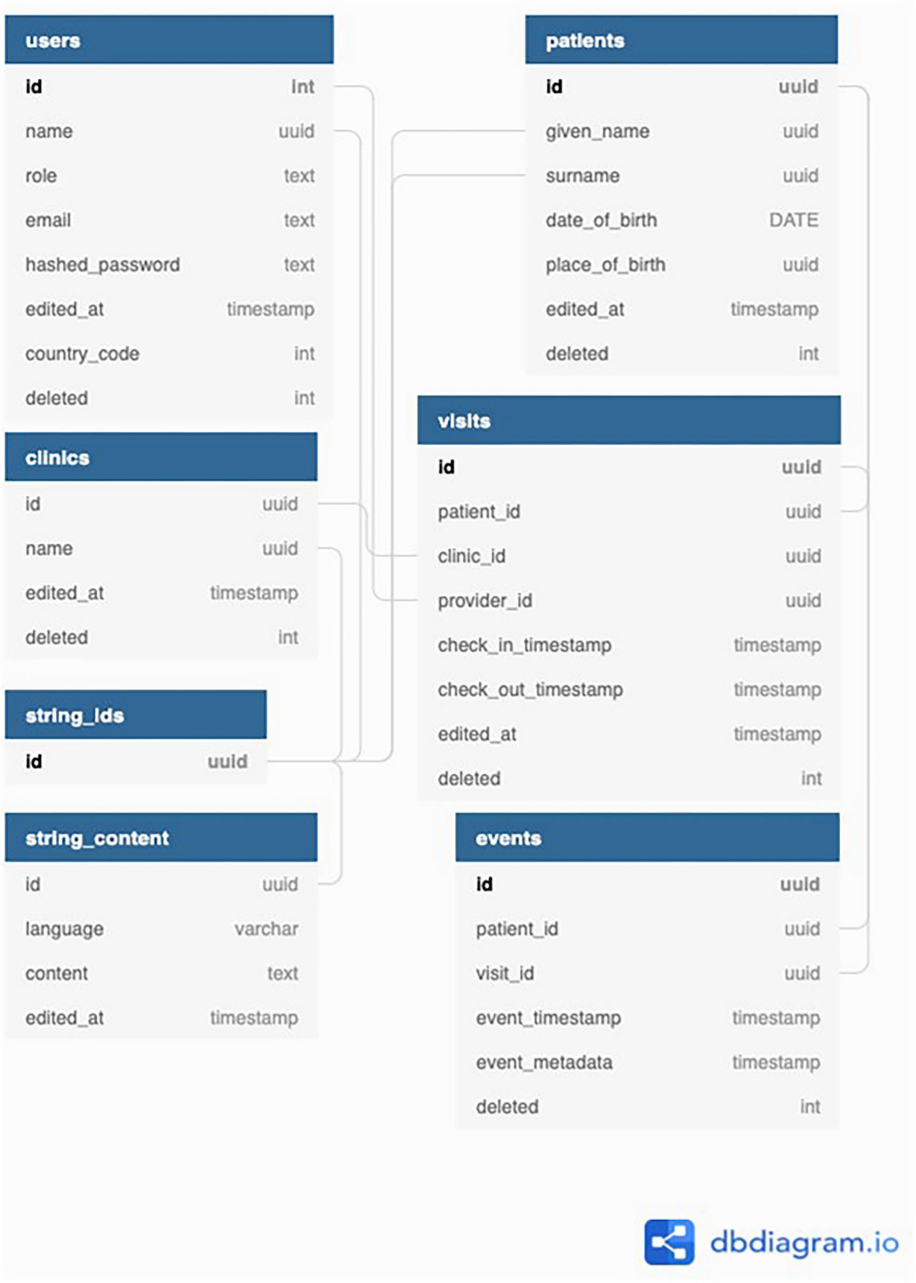
A diagram demonstrating how the sync architecture within the EHR system from the front end.

Both applications connect to the same *Python backend repository* that sits on a PostgreSQL Database (Figure 3), which is deployed on Google Cloud SQL using Kubernetes and Docker. The repository is home to a mobile API and a photos API that handle the login and sync functionality detailed in the next section. An admin API is also present to handle requests from the admin app related to user management and exporting patient data.

### Sync Architecture

**Figure 4:**
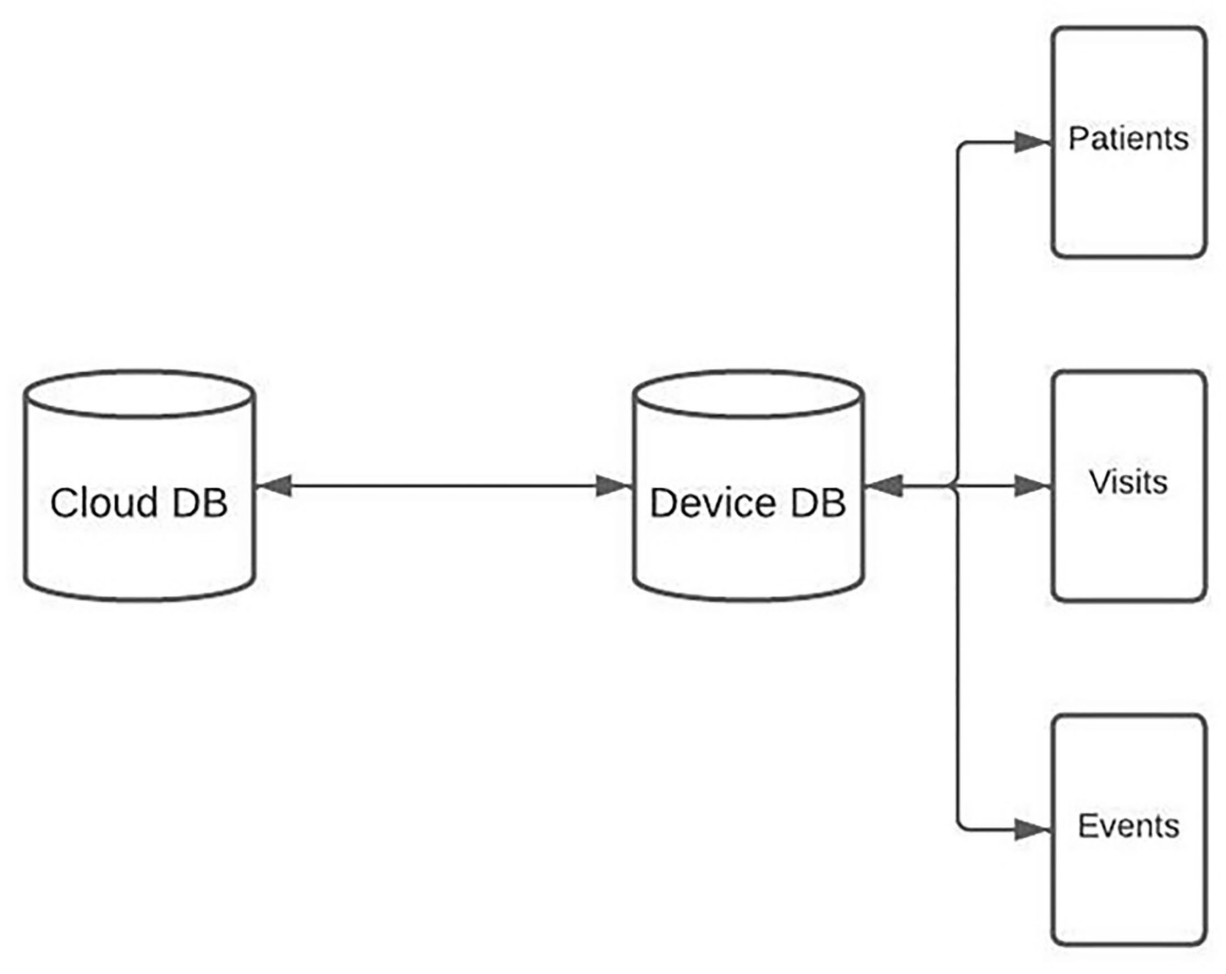
A diagram depicting the Hikma Health database schema.

Every device running the app has a SQLite database that is used to create, read, and update patient information on the device. The schema for this database is identical to the central PostgreSQL database (DB) which is deployed on GCP using Kubernetes and Docker. Deletions of patient records are handled by marking a field as deleted = TRUE. Medical records can be added and updated in the app throughout the day. When the medical provider gets data service, the device level database can be synced with the database on the central cloud based server (Figure 4).

On sync, RNFS and RNFetchBlob are used to POST the entire device level database to the sync API, which is built with Python. All database rows have a timestamp field, and IDs and timestamps are queried for all client tables and compared with the corresponding table in the server database. For rows that are missing or outdated on the server, SQL is generated and run to create or update the entity on the server. If rows are missing or outdated on the client database, client SQL is generated to run on the device. Client SQL is sent to the device as a JSON response of the sync HTTP request, asynchronously run to create or update the entity on the device.

### Photo Sync Architecture

Syncing the DB also initiates the sync of patient images. First, patient image metadata is retrieved from the server. This metadata includes the patient ID and filename for each image that is stored in a GCS bucket. The filename is the timestamp from when the image is captured. A list is compiled that contains patient IDs for which a photo is stored on the server, but not on the device. Another list is compiled that contains patient IDs for which a photo is stored on the device, but not on the server. For the intersection of those lists, timestamps are compared to see if the image needs to be updated either on the server or the device.

For all the photos that must be added or updated on the server, RNFetchBlob is used to send the photo to the server, which stores the photo on the GCS bucket and updates the filename on the patient record to be the updated timestamp. For the photos that must be added or updated on the device, the image timestamp is updated on the device, and the photo is retrieved from the GCS bucket using RNFetchBlob. RNFS is then used to write the image file to the local photo directory and organized by patient ID. This process takes a few seconds, so it executes asynchronously and does not disrupt user workflows.

## Results

### Implementation in Lebanon

The Hikma Health system was initially designed for use by Endless Medical Advantage (EMA), a non-profit organization operating mobile, van based clinics to provide care to thousands of refugee patients in the Bekaa Valley of Lebanon. EMA first began using the Hikma Health system in early 2020 and has since consistently used the application while treating patients to collect and access patient data. Improvements to the Hikma Health system have been made through continuous conversations with EMA staff and clinicians based on their evolving clinical needs. Since initial deployment, the EMA version of the Hikma Health system has significantly evolved and improved in both the design and features. The April 2020 update included a screening tool for COVID-19 that implemented logic outlined by the WHO and CDC screening workflows.

**Figure 5:**
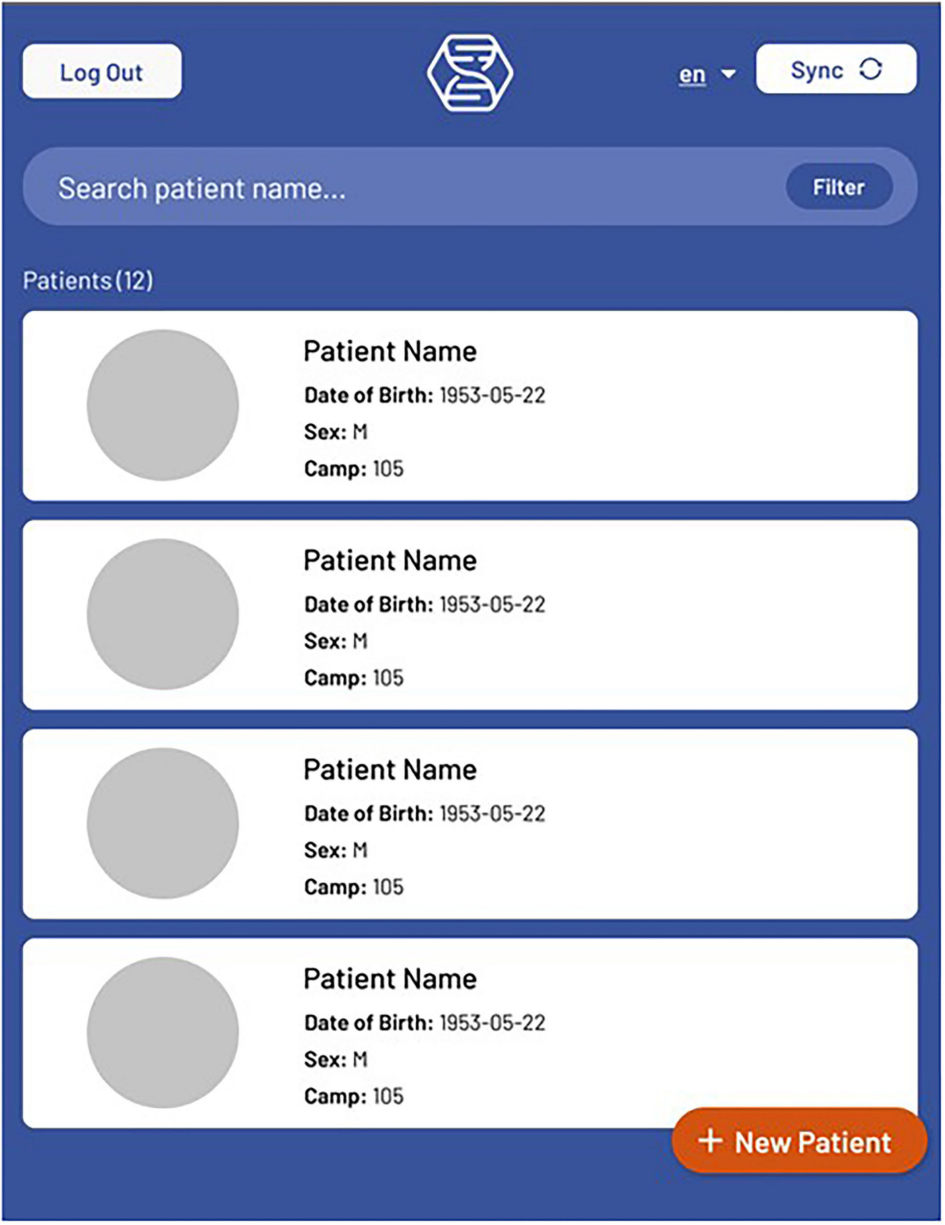
The Hikma Health updated search function allows for patients with similar characteristics to be searched.

After clinicians' initial use of the Hikma Health system in the field, they reported two main usability issues: they were having difficulty successfully finding previous patients using the patient search tool and were unaware if syncing data was successful. In the first major update to the Hikma Health system both of these concerns were addressed seen in Figure 5. To address the challenges clinicians were having with the search tool, additional fields were added to the search queries beyond name, including country, hometown, camp number, phone number, and date of birth. Users reported positive feedback on the advanced search feature, but still encountered challenges searching for patients because of misspelled names, particularly due to multiple valid transliterations of many Arabic names into Latin characters. To improve the search capabilities, a fuzzy search feature was added, which returned results including patients whose names were a differently spelled version of the searched name, for the 15 most common patient names in the region that had multiple transliterations. For example, searching “Mohammed” would also return a patient named “Muhammad” in the search results. A notification was added to inform the user on the success or failure of the data sync.

Another issue clinician users reported was the need to delete visits that were added mistakenly or with incorrect information. Deleting rows in the database is not a viable solution for this problem, given the nature of the sync architecture. Therefore, a Boolean deleted field was added to the visit table, enabling users to mark visits as deleted, removing them from all displays.

The patient view screen has undergone several major design updates. The first update included an improvement to the look and feel of the screen, the addition of the patient's camp, and a link to a new prescription list page from which medicines could be viewed and edited. The next significant update was the addition of a patient snapshot feature, where previous events (i.e., completed forms) could be viewed by the type of event. Prior to this feature, patient events were only viewable by navigating to the visit history for a patient and selecting the visit during which the given event occurred. The snapshot allowed medical providers to more easily find pertinent patient information.

Users often added multiple visits in the application while caring for a patient in a single session, because events could only be added to new visits. An update was added that allowed users to add events to previous visits, so that information could be more accurately grouped. The location of the patient camp event input was also moved to the add and edit patient screens, since this event type was being used as a detail for identifying patients, unlike the other visit events. All screens of the application underwent a style update to optimize the view for bigger screens, since tablets are the preferred tools for most users.

A review of our Covid-19 screening tool was performed by the Hikma Health medical team, and the results showed that the existing algorithm used to recommend treatment needed to be updated to reflect the current situation of the pandemic. Advice for seeking emergency care was implemented, which was recommended for patients presenting with symptoms of chest pain, confusion, or bluish lips/face. Suggestions of testing and isolating were made for other less severe symptoms of Covid-19. The navigation of the workflow was also improved to reduce the required number of user taps.

Testing and quality control of the application was made easier by adding patient and visit counts to list screens, so that these counts could be compared with corresponding counts in the exported data. Event displays and input forms were styled for a better user experience and to increase clarity of event type.

Hikma Health managed the deployment of the Hikma Health system in Lebanon for over 2 years. Through regular check-ins the EMA clinical and administrative staff report that the system has enabled them to increase efficiency and quality of care compared to their previous paper-based recordkeeping system. In January 2022, Hikma Health supported EMA in successfully transitioning the management of the deployment of the system to an in-house technology team. EMA's independent management will enable the deployment of the system to be sustainable, allowing EMA to further develop and refine the technology to be further customized for their unique context and requirements.

### Implementation in Nicaragua

In 2020, Hikma Health partnered with the Nueva Vida (NV) Clinic, a clinic based in Ciudad Sandino, Nicaragua serving thousands of low-income rural patients. Hikma Health worked with clinical staff to identify the recordkeeping and documentation practices of NV, which were considerably more involved than EMA's. A manuscript for the full stack implementation of new forms is provided to each clinic as one of the first steps of onboarding for the app. Through a needs assessment, Hikma Health developed a new version of the Hikma Health system specifically for the NV, which included additional features and 21 new visit event forms. Additionally, while the visit event forms for EMA consisted largely of text input fields, the forms for NV all have a high quantity of more complex inputs.

The implementation of the NV version of the Hikma Health system involved fewer iterations than the initial version. Feedback for EHR improvements were gathered through annual process improvement meetings and not through formalized interviews or surveys. For each of the new event forms needed for NV, the work consisted of building out the front-end components for the input form and display, providing links to these components in multiple screens, and updating the export functionality to include the new information in the exported spreadsheet. While implementing these steps is a notable amount of work, forms can be added and changed without database migrations being necessary, since event metadata is saved in the database as JSON strings. Thus, the Hikma Health system can easily be adapted to suit new clinics. As of publication, NV's technology team has been successfully managing the deployment of the Hikma Health system for 6 months within the clinic.

## Discussion

To date, the Hikma Health system has positively impacted care for 26,000 patients in Lebanon and Nicaragua. The positive impacts of the system reported by clinician and clinical administrators through regular feedback meetings are 3-fold: (1) improved continuity of care; (2) improved visualization of clinical data; and (3) improved efficiency resulting in a higher volume of patients. Continuity of care is improved by clinicians having reliable access to the entire patient record on any device. Users report that clinical data is more easily visualized in the Hikma Health system than in the handwritten records or spreadsheets that were previously used. Clinical leadership has also reported that overall they have observed increased efficiencies in clinical operations, enabling them to care for a larger patient volume. In the future, we aim to quantitatively assess with our clinical partners these subjective observations of improved care practice by staff.

A major adaptation that was made early in the implementation of the EMA application involved the “refugee camp” field. This field was initially built as a type of visit event, but it soon became clear that it would be used primarily as a patient identification field. Therefore, the modification to an identification field was necessary to save this field as part of the patient registration process and include it in the patient search. In future versions of the Hikma Health system, the camp field could be altered to be used as neighborhood, region, or similar geographical identification, depending on the needs of the clinic. The importance of synchronization confirmation was a significant lesson learned during the EHR implementation. In early 2021, an edge case existed that caused the sync functionality to be temporarily broken. The medical providers at EMA were unaware that the sync was failing, so the issue went undetected for several days. It became apparent that notification of either success or failure of attempted synchronization was necessary to inform clinician users, and this functionality was included in later versions.

### Limitations

While built for use in primary care, urgent care, or multi-specialty clinics, the Hikma Health system is currently limited in supporting clinical workflows involving significant diagnostic data, such as those found at tertiary hospitals. It does not yet support importing vitals, labs, or advanced imaging (e.g., DICOM) directly into the system. Future development cycles will address these capabilities to further support advanced diagnostics as related hardware technologies become more globally accessible. Due to the settings in which it was primarily developed for use in, Hikma Health system does not include modules for billing, scheduling, or general enterprise resource planning. We would also like to note that the data we gather has been through a continual iterative process with our partners and not a systematic investigation.

## Conclusion

The Hikma Health system presents a novel, offline-first, data system for the care of patients in low resource, low connectivity settings. The Hikma Health system has proven to be particularly well-suited for refugee and migrant patients who currently suffer from discontinuous and irregular access to care. Appropriately provisioned mobile devices are used by clinician users to read and write data about patients locally to be securely stored immediately and synced to the central database once connectivity is established. The system is fully available in English, Arabic, and Spanish to enable multi-lingual care teams that often practice in humanitarian settings. Finally, the highly customizable open-source architecture lends itself to future iterative development to support a growing range of clinical workflows and use cases. As the number of displaced people continues to rise, it is vital that solutions like the Hikma Health EHR are developed to improve the care provided and patient outcomes.

## Restrictions for Use

The Hikma Health system is available online for anyone to download and modify without any restrictions under the MIT License. We welcome anyone to use and modify our technology to meet their needs.

## Data Availability Statement

The original contributions presented in the study are included in the article/supplementary material, further inquiries can be directed to the corresponding author.

## Ethics Statement

Ethical review and approval was not required for the study on human participants in accordance with the local legislation and institutional requirements. The patients/participants provided their written informed consent to participate in this study.

## Author Contributions

HA drafted the initial outline and wrote the introduction. TB provided the technical writing and analysis. HA, SB, SE, AK, and HE wrote the results and conclusion. All authors reviewed the final manuscript, conceived of the article, and were involved in initial drafting. All authors contributed to the article and approved the submitted version.

## Funding

The Hikma Health EHR was developed with financial support from Fast Forward, MassChallenge, the Gerson Lehrman Group, HP Enterprise, Google.org, BlackRock, the Harvard Business School New Ventures Competition, the Harvard Arab Alumni Association, the MIT $100K Competition, the MIT Media Lab, the MIT Sandbox Innovation Fund Program, and the Robert Wood Johnson Foundation.

## Conflict of Interest

TB is a paid technology consultant for Hikma Health Inc., a 501(c)(3) non-profit organization. SB was formerly a paid technology consultant in 2019–2020 for Hikma Health Inc, and is currently a volunteer. The remaining authors on this manuscript are volunteers for Hikma Health Inc. and have not received any financial compensation for their work.

## Publisher's Note

All claims expressed in this article are solely those of the authors and do not necessarily represent those of their affiliated organizations, or those of the publisher, the editors and the reviewers. Any product that may be evaluated in this article, or claim that may be made by its manufacturer, is not guaranteed or endorsed by the publisher.

## Abbreviations

UNHCR: United Nations High Commissioner for Refugees
HER: Electronic Health Record
GCP: Google Cloud Platform
GCS: Google Cloud Storage
SQL: Structured Query Language
JSON: JavaScript Object Notation
API: Application Programming Interface
HTTP: Hypertext Transfer Protocol
EMA: Endless Medical Advantage
NV: Nueva Vida
RNFS: React Native File System
RNFetchBlob: React Native Fetch Blob.
